# The Maternal Brain: Investigating effects of Parity on Brain Structure and Risk for Dementia in Postmenopausal Women

**DOI:** 10.1002/alz70856_100523

**Published:** 2025-12-25

**Authors:** Isabella Tucci, Colleen Lacey, Jodie Gawryluk

**Affiliations:** ^1^ University of Victoria, Victoria, BC, Canada

## Abstract

**Background:**

Alzheimer's disease (AD) is more prevalent in women than men, with female sex being a major risk factor. Emerging evidence suggests that estrogen exposure from female‐specific factors may contribute to this increased risk. However, the relationship between parity and hippocampal grey matter volume (GMV) remains underexplored, despite pregnancy‐induced hormonal changes and the hippocampus’ early vulnerability to neurodegeneration. This study examined the association between parity, hippocampal GMV, and cognitive function in postmenopausal women.

**Method:**

Data were drawn from the UK Biobank. Female participants with T1‐weighted MRI and complete demographic, lifestyle, and reproductive data were included (*n* = 1659). To ensure a cognitively healthy sample, those diagnosed with mild cognitive impairment (MCI) or dementia were excluded, yielding a final sample of 1284 postmenopausal women. Multiple linear regression analyses examined the relationship between number of live births and three outcome variables: left and right hippocampal GMV and errors on the Pairs Matching Test (a visual memory task). Covariates included age, ethnicity education, BMI, and total brain volume. Parity was categorized as 0, 1, 2, 3, 4, or ≥5, with nulliparous women as the reference group.

**Result:**

Multiple linear regression analyses revealed having one child was significantly associated with lower right hippocampal GMV (β = ‐83.89, *p* =  0.0169) and left hippocampal GMV (β = ‐83.06, *p* =  0.0144) compared to nulliparous women. Similarly, having one child was linked to poorer memory performance, as indicated by a higher number of errors on the Pairs Matching Test (β = 0.60, *p* =  0.049). No significant differences were observed for women with two or more children.

**Conclusion:**

These preliminary findings suggest that having one child may be linked to reduced hippocampal GMV and poorer memory in postmenopausal women, whereas having multiple children was not associated with these outcomes. Given the hippocampus' role in memory and AD pathology, parity may be a factor in women's heightened AD risk. Future analyses will explore how hormone replacement therapy (HRT) and loneliness modulate these relationships, providing deeper insight into cognitive aging and AD vulnerability in women.

